# The Impact of Protein Acetylation/Deacetylation on Systemic Lupus Erythematosus

**DOI:** 10.3390/ijms19124007

**Published:** 2018-12-12

**Authors:** Jingjing Ren, Eric Panther, Xiaofeng Liao, Amrie C. Grammer, Peter E. Lipsky, Chris M. Reilly

**Affiliations:** 1 Department of Biomedical Sciences and Pathobiology, Virginia-Maryland College of Veterinary Medicine, Virginia Polytechnic Institute and State University, Blacksburg, VA 24060, USA; renjj@vt.edu (J.R.); epanther@vt.edu (E.P.); 2 Edward Via College of Osteopathic Medicine, Blacksburg, VA 24060, USA; 3 Department of Biological Sciences, Virginia Polytechnic Institute and State University, Blacksburg, VA 24060, USA; xifeli@vt.edu; 4 AMPEL Bio Solutions, 250 West Main Street, Charlottesville, VA 22902, USA; amriegrammer@comcast.net (A.C.G.); peterlipsky@comcast.net (P.E.L.)

**Keywords:** systemic lupus erythematosus, lupus, methylation, acetylation, histone deacetylase inhibition

## Abstract

Systemic lupus erythematosus (SLE) is a chronic inflammatory autoimmune disease in which the body’s immune system mistakenly attacks healthy cells. Although the exact cause of SLE has not been identified, it is clear that both genetics and environmental factors trigger the disease. Identical twins have a 24% chance of getting lupus disease if the other one is affected. Internal factors such as female gender and sex hormones, the major histocompatibility complex (MHC) locus and other genetic polymorphisms have been shown to affect SLE, as well as external, environmental influences such as sunlight exposure, smoking, vitamin D deficiency, and certain infections. Several studies have reported and proposed multiple associations between the alteration of the epigenome and the pathogenesis of autoimmune disease. Epigenetic factors contributing to SLE include microRNAs, DNA methylation status, and the acetylation/deacetylation of histone proteins. Additionally, the acetylation of non-histone proteins can also influence cellular function. A better understanding of non-genomic factors that regulate SLE will provide insight into the mechanisms that initiate and facilitate disease and also contribute to the development of novel therapeutics that can specifically target pathogenic molecular pathways.

## 1. Introduction

Systemic lupus erythematosus (SLE) is a pathophysiologically complex systemic autoimmune disease affecting multiple organs [1,2,3]. Epigenetic regulation refers to the change of the epigenomic pattern that in turn alters gene expression specifically related to modified DNA sequences [4]. Epigenetic abnormalities in cancer research has been widely studied for decades. Because disease pathogenesis between autoimmune diseases and cancer share some similarities, epigenetic contributions have been proposed in the regulation of autoimmune disease [5]. Epigenetic regulation may involve microRNAs, acetylation and methylation of histone proteins and DNA methylation—all of which have been linked to the initiation, onset, progression and perpetuation of SLE [6,7,8]. Attenuation of lupus-like disease in murine models via treatment with histone modifiers has been reported in many studies. In regard to histone acetylation/deacetylation, different isoforms of histone deacetylase enzymes may be predominantly nuclear, cytoplasmic or shuttle between the nucleus and cytoplasm. Our studies have demonstrated that histone deacetylases (HDACs) are significantly upregulated in lymphocytes in MRL/lpr lupus prone mice [9,10]. Other studies have also found elevated histone acetylation in innate immune cells, such as monocytes [11,12,13]. Moreover, studies have shown that global alteration of DNA methylation is pathogenic in lymphocytes and innate immune cells; specifically, hypomethylation has been highly correlated with disease activities in SLE patients [14]. In this review, we will briefly summarize the role of DNA methylation in SLE and highlight immunopathogenic contributions of acetylation and deacetylation to lupus. 

## 2. Systemic Lupus Erythematosus

Systemic lupus erythematosus (SLE) is a multifactorial autoimmune disease that involves genetic predisposition, epigenetic modification and environmental factors that lead to alteration in both the innate and adaptive immune responses, including abnormalities in apoptotic cell clearance, cytokine production, and dendritic cell, B-cell and T-cell activation [1,15,16]. Lupus is highly associated with deficiency of apoptotic clearance [17]. While the initial triggers vary, the excessive accumulation of apoptotic debris, in particular microparticles containing nuclear material, can activate antigen presenting cells including dendritic cells and B cells, which initiate the cellular interactions that lead to the generation of antinuclear antibodies through interactions with autoreactive T cells [17,18]. T cells not only activate B-cell responses but also infiltrate target tissues and cause damage. Type I and type II interferons (TFN I/II), tumor necrosis factors (TNF), B-lymphocyte stimulators (BLys), interleukin 6, interleukin 17, interleukin 18, interleukin 21, and many other cytokines are involved in autoimmune priming and induce inflammatory mediated tissue injury in patients with lupus [19,20,21].

One of the most severe manifestations of SLE is lupus nephritis (LN). LN remains a major cause for morbidity and mortality in SLE patients [3,22,23,24,25,26,27,28]. Glomerulonephritis (GN) is the most common form of LN, which is frequently accompanied by tubulointerstitial and/or vascular lesions [29]. In the kidney, immune complexes containing anti-DNA and anti-nucleosome antibodies contribute to lupus nephritis and initially deposit in the endothelial and mesangial areas, and then in the basement membrane and epithelial areas [30]. Importantly, these immune complexes initiate a further influx of inflammatory cells by activating the complement cascade.

Genetic variations suggest a predisposition to SLE development [31]. Both single gene deficiency, such as complement C1q and C4, and the effect of a large number of genetic variations including single-nucleotide polymorphisms (SNPs) within noncoding regions of immune response–related genes are associated with SLE [31,32,33]. However, these findings can only account for about 15% of the heritability of SLE, suggesting that factors other than genetic variations may have additional effects on SLE development [34]. UV light exposure is a well-documented environmental trigger of SLE [2,29]. However, paradoxically, avoidance of sunlight to reduce UV light exposure can result in a vitamin D deficiency, which is negatively associated with SLE severity [35]. Additionally, viral infections may trigger SLE by self-antigen mimicry and induction of inflammation [36,37,38]. As the disease ratio of female to male is about 9 to 1, hormones and the X chromosome are believed to play a role in the increased prevalence of SLE among women [39,40]. Epigenetic changes, including DNA methylation, histone modifications (acetylation and methylation), and acetylation of non-histone proteins, influence DNA accessibility to transcription factors, as well as translocation and addition of proteins resulting in altered gene expression. These epigenetic changes have emerged as possible mechanisms that initiate or contribute to the pathology of SLE.

## 3. Methylation in SLE

**Figure 1:**
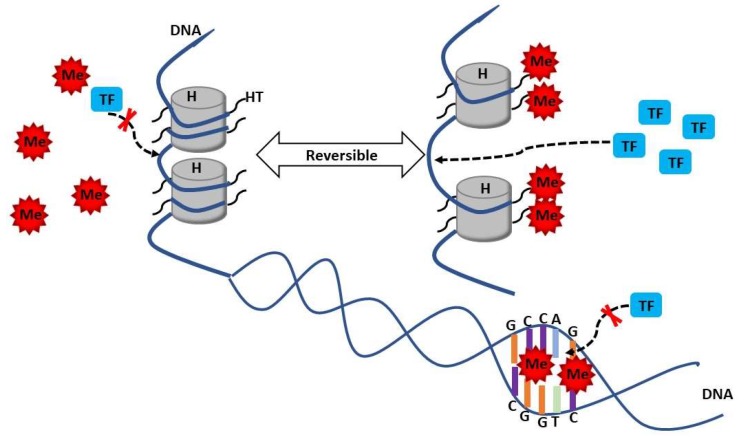
Histone and DNA methylation and demethylation. Histone methylation adds methyl group to the residue of histone tails and increases gene transcription by uncoiling DNA from histone and opening more DNA binding sites to transcriptional factors. DNA methylation happens between paired CG groups in DNA sequences. It prohibits the transcriptional factors binding to DNA and represses the gene transcription. H: Histone; HT: Histone tails; Me: Methylation; TF: Transcriptional factor.

Methylation of C bases in CG pairs, known as DNA methylation, is characterized by the addition of a methyl group to the carbon residue in a gene promoter region. Histones are a group of conserved proteins associated with the DNA strand that regulate DNA stability, replication, and transcription through different histone modifications, including methylation and acetylation. The modification of DNA and histones may act as epigenetic regulators of gene expression. DNA methylation promotes the repression of gene transcription through prevention of transcription factors binding to the chromatin structure [41]. Histone modifications can be very dynamic based on the activation and differentiation status of the cell (Figure 1) [42,43].

The induction of this lupus-like syndrome depends on the level of methylation of genome DNA from the apoptotic cells. Studies have reported that perturbation of DNA methylation results in the activation of the apoptotic pathway. Aberrant clearance of apoptotic DNA plays a critical role in the development of SLE. It has also been shown that the injection of extracted DNA from stimulation-induced apoptotic lymphocytes into a mouse model induced a lupus-like disease, characterized by the production of anti-dsDNA antibodies and lupus nephritis [44,45,46]. Upon re-methylation of apoptotic DNA, the ability to induce anti-dsDNA antibody was dampened. In contrast, an increase in DNA demethylation boosts its ability to stimulate an autoimmune response [47]. Toll-like receptor 9 (TLR9), which recognizes hypomethylated DNA to induce an inflammatory response in innate immune cells, drives an enhanced type I interferon response that is likely to contribute to the DNA methylation effect on SLE development [48]. 

Studies have used DNA methylation inhibitors to induce lupus-like diseases in murine models and found impaired DNA methylation in SLE patients, both of which support the relationship between DNA hypomethylation and lupus disease [49,50,51]. CD11a is an integrin involved in cellular adhesion and co-stimulation and provides a critical initial interaction between T cells and antigen-presenting cells, stabilizing the immune synapse. CD11a is overexpressed in lupus via the regulation of DNA methylation. Lu et al. show that overexpression of CD11a from CD4+ T cells in patients with active lupus is positively correlated with SLE disease activity index (SLEDAI) score. They further demonstrated that demethylation of the promoter region of CD11a is higher in active lupus patients compared with inactive patients and normal controls [52].

Enhancer of Zeste homologue2 (EZH2) can act as an epigenetic regulator of gene expression, either through trimethylation of lysine 37 in histone 3 (H3K27me3) or direct control of DNA methylation by recruiting DNA methyltransferase [53,54]. Unlike other histone methylation, which relaxes chromatin and turns on gene transcription, trimethylation of H3K27 causes the chromatin to compact and repress gene transcription [55,56,57]. In lupus patients, H3K27me3 is enriched in the hematopoietic progenitor kinase 1 (HPK1) promotor region [58,59]. As HPK1 acts as a negative regulator of T cell-mediated IFNγ and IgG production, it may explain part of the lupus pathophysiology. Additionally, EZH2 is highly expressed in naïve CD4+ T cells in lupus patients compared to healthy controls and positively correlates with lupus disease activity. Increased EZH2 expression also induces overexpression of junctional adhesion molecule A (JAM-A) through DNA hypomethylation. In this regard, upregulated JAM-A promotes T cell adhesion and T cell survival, increasing the ability for T cells to migrate to inflamed tissues in lupus [60]. 

CD40L (CD154) and CD70 are two molecules that are expressed on activated T cells and involved in co-stimulation during T cell: B cell interaction. Both molecules have been shown to be upregulated on T cells in patients with active SLE compared to lupus patients with inactive disease or healthy individuals. Furthermore, hypomethylation of these molecules at the promoter regions of T cells has been demonstrated to promote disease [61,62,63]. Recently, a study by Ulff-Muller and coworkers reported that hypermethylated DNA in B cells contributed to SLE, which contrasts with DNA hypomethylation in T cells [64]. In addition to methylation of DNA, acetylation of histone and non-histone proteins has been reported to play a role in the development of lupus and has been of particular interest in our research studies [65].

## 4. Acetylation in SLE

**Figure 2:**
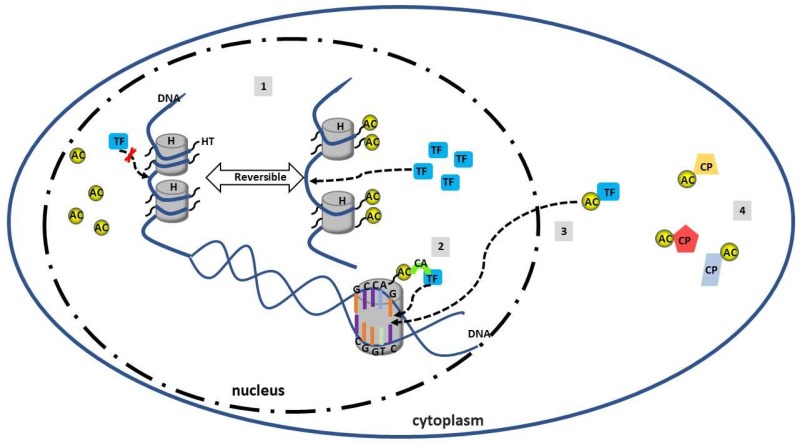
Histone and nonhistone protein modified by acetylation and deacetylation. Acetylation and deacetylation refer to removal or addition of acetyl group from targeted protein or DNA. (**1**) Histone acetylation and deacetylation is a dynamic and reversible reaction that alters the structure of histones and affect the gene transcription by loosening or compacting the DNA. (**2**) Acetylated histones can act as a binding site for other proteins which are co-activators of transcriptional factors. (**3**) Nucleus translocation and DNA binding affinity of transcriptional factors in the cytoplasm can be modified by acetylation or deacetylation. (**4**) Acetylation and deacetylation can regulate other nonhistone proteins in the cytoplasm and alter their function in cellular activities. H: Histone; HT: Histone tails; AC: Acetylation; TF: Transcriptional factor; CA: Co-activator; CP: Cytoplasm protein.

The acetylation and deacetylation of amino-acid residues within histone tails has been shown to be a main factor influencing chromatin structure and modulating gene transcription, both positively and negatively [66,67]. Histone acetylation regulates gene transcription in different ways. First, acetylation of lysine residues within histone tails neutralizes the positive charge of histone proteins, loosening chromatin structure. This increases the accessibility of transcription factors to the promoter regions of their target genes [68]. Second, acetylated histones also function as binding sites for other proteins that act as transcriptional co-activators. In contrast, histone deacetylation promotes transcriptional suppression via chromatin compaction [69]. Third, direct acetylation and deacetylation of transcription factors and proteins, other than histones, have been shown to have both positive or negative regulating roles on gene expression (Figure 2) [70].

Histone acetylation is controlled by the opposite actions of two large families of enzymes—the histone acetyltransferases (HATs) and histone deacetylases (HDACs).

The HDAC superfamily encodes 11 proteins with a highly conserved deacetylase domain (Table 1). These proteins can be classified into four families (class I, IIa, IIb and IV), which differ in function, cellular and subcellular localization, and expression patterns. The class I HDAC family consists of HDAC1, 2, 3 and 8 [71,72,73]. They are expressed ubiquitously, localized predominantly to the nucleus, and display high enzymatic activity toward histone substrates. HDAC1, HDAC2 and HDAC3 are nearly identically in repressive complexes [74], whereas HDAC8 seems to work alone without binding to repressive complexes [72].

HDAC4, 5, 7 and 9 belong to the class IIa HDAC family. They have conserved binding sites for the transcription factor myocyte enhancer factor 2 (MEF2) and the chaperone protein 14-3-3. These HDACs can translocate between the nucleus and the cytoplasm after phosphorylation by kinases and thereafter binding to the chaperone protein 14-3-3 [75,76,77,78]. The regulated phosphorylation of class IIa HDACs functions as a linker between extracellular signals and transcriptional changes in the nucleus, and plays key roles in different tissues during development and disease. Different from other HDACs, class IIa HDACs show relatively restricted expression patterns, with HDAC4 expressed in the brain, HDAC5 and HDAC9 enriched in muscle, the heart and brain, and HDAC7 mostly found in the thymus and endothelial cells [75,79,80,81].

The Class IIb family consists of HDAC6 and HDAC10. HDAC6 mainly resides in the cytoplasm, and the function of HDAC6 is not well understood [82,83,84]. The direct targets of HDAC6 include the cytoskeletal proteins α-tubulin and cortactin, transmembrane proteins such as the interferon receptor IFNαR, and chaperones [85,86,87,88,89]. HDAC11 is the only class IV HDAC discovered to date, but its function is not well delineated [90,91].

The roles of HDACs in lupus have been reported by us and others, and HDAC inhibitors are indicated to be a possible new treatment for lupus. There are pan-HDACs and selective HDAC inhibitors. Pan-HDAC inhibition causes a global interference of epigenetic programming which could result in both therapeutic as well as adverse effects. In contrast, selective HDAC inhibition has less toxicity based on their limited substrates, location, and specific enzyme activities.

In T cells of SLE patients, CD40L and IL-10 are overexpressed, whereas IFN-gamma is downregulated [104]. After treatment with trichostatin A (TSA), a reversible pan inhibitor of HDAC, the expression levels of CD40L, IL-10 and IFN-gamma were corrected in patient T cells, suggesting the involvement of HDACs in abnormal T cell activation in SLE patients through at least regulating CD40L and IL-10 expression, which are both critically involved in T cell: B cell interaction and B cell activation. TSA also suppressed IL-2 production by activated T cells by downregulating T cell receptor zeta chain signaling [105]. Treatment of either TSA or another HDAC inhibitor, suberonylanilide hydroxamic acid (SAHA) targeting HDAC I in MRL/lpr lupus-prone mice showed reduced proteinuria, glomerulonephritis, spleen weight, and mesangial cell inflammation, associated with an increased accumulation of acetylated histones H3 and H4 in total cellular chromatin [106,107]. Recently Hu et al. reported reduced H3 and H4 acetylation and decreased IFN-gamma expression in mouse splenocytes of SLE patient peripheral blood mononuclear cell (PBMC) [108]. Conversely, Garcia et al. reported hypoacetylation in splenocytes of MRL/lpr lupus-prone mice compared to MRL/MpJ control mice [109]. Besides the effects of HDACs on T cell activation, it has been reported that HDAC1 is recruited to the IgH enhancer region, and TSA treatment of B cells reduced the production of anti-DNA autoantibodies directly, highlighting the influence of HDACs on B cells in lupus mice [110]. In New Zealand Black/White (NZB/W) F1 female mice, TSA administration resulted in an increase in regulatory T cells and a decrease of CD69+ activated T helper cells which correlated with reduced lupus nephritis [111]. Another pan-HDAC inhibitor, panobinostat targeting class I, II and IV HDACs, decreased the percentage of autoreactive plasma cells and reduced the production of autoantibodies in MRL/lpr mice in pre-disease stage [112].

Hu et al. reported differential functions and expression patterns of different HDACs in MRL/lpr mice. They demonstrated increased SIRT1 expression but decreased HDAC7 expression in MRL/lpr mice compared to MRL control mice [113]. SIRT1 siRNA treatment reduced tubulointerstitial scores but had no effect on proteinuria and serum autoantibody levels, which was different from the effects of the pan HDAC inhibitor TSA. HDAC3 and 11 have been reported to be decreased in monocytes from SLE patients compared to healthy individuals, suggesting their possible roles in suppressing autoimmune responses [114]. In addition, the transcription factor RFX1 which recruits the co-repressor HDAC1 is reduced in T cells of SLE patients. This results in the overexpression of CD11a and CD70 on T cells of SLE patients, suggesting the immunosuppressive effect of HDAC1 and suggests the immune repressive effect of TSA may work on HDACs other than HDAC1 [115]. HDAC9 deficiency in MRL/lpr mice has been shown to reduce lupus symptoms and increase survival rates compared to HDAC9 intact MRL/lpr mice [116]. In the study, effector T cells in the HDAC9-/- mice switched from a Th1 and Tfh into a Th2 phenotype with increased acetylation of histone proteins globally at the IL-4 gene locus, suggesting HDAC9 inhibition may benefit SLE patients. However, TSA is a pan HDAC inhibitor. Another study, using a selective class I and II HDAC inhibitor (ITF2357) demonstrated reduced disease in NZB/W F1 mice, suggesting that class I and II HDAC are involved in lupus pathogenesis [117]. In addition to regulating protein-translating genes, HDACs have also been reported to regulate microRNAs that suppress B cell responses [118]. We have recently reported that HDAC6 inhibition upregulated microRNA targeting AID and Blimp-1, which are critical factors in B cell responses, resulting in reduced lupus disease in MRL/lpr mice. Furthermore, we found, reduced germinal center B cells, T follicular cells and IFN-gamma secreting cells suggesting HDAC6 inhibition contributed to the downregulation of adaptive immune response in lupus nephritis [119]. In our studies, we have shown that selective HDAC6 inhibition decreases IFN-alpha production in initiation stage of the disease. Furthermore, we found that administration of the selective HDAC6 inhibitor ACY-738 in vitro led to decreased IFN-alpha production in a dose-dependent manner [119]. HDACs also promote neutrophil extracellular traps (NETs) which act as an important source of self-antigens [120]. Our previous studies have shown MRL/lpr mice have increased levels of HDAC6 and HDAC9 compared to non-autoimmune B6 mice. However, B6 animals showed increased expression of HDAC10 compared to MLR/lpr lupus prone mice in splenic B and T cells [9]. As HDAC6 is primarily localized to the cytosol, the selective HDAC6i, ACY-738, blocks HDAC activity in the cytoplasm while not affecting nuclear histones [121]. We found that HDAC6i treatment of B cells increased NFkB acetylation, prevented translocation to the nucleus, and suppressed B cell development at the pre-B cell stage [122]. Further treatment with ACY-738 in NZB/W F1 mice reduced lupus nephritis, sera anti-dsDNA level associated with increased splenic Tregs, and decreased Th17 cells [103,122]. These studies suggest that HDAC6 is involved in lupus development by various mechanisms. Transcription factor Fli-1 regulates G-CSF production to control neutrophil infiltration into the kidneys, causing kidney inflammation in lupus [123,124,125]. Deacetylation at aa380 decreases Fli-1 driven activation of the G-CSF promoter to decrease inflammatory cytokine secretion in lupus prone mice [126]. In human studies, Th17 cells play a pivotal role in the contribution to the pathogenesis of SLE via secreting the IL-17 inflammatory cytokine. Increasing HDAC3 acetylation led to overexpression of IL-17A through downregulating expression of transcription factor RFX1, which suggests HDAC3 acts as a nuclear epigenetic regulator in SLE patients [127].

## 5. Metabolism and Epigenetic Crosstalk in Lupus

The methylation/demethylation of DNA and histones and acetylation/deacetylation of histone and nonhistone proteins alter gene expression and immune cell function in lupus. However, these epigenetic reactions are reversible and can be affected by the availability of substrates from metabolic pathways. Advancements in the field of immunometabolism have suggested that aberrant metabolic pathways may also play a critical role in the pathogenesis of lupus disease [128].

Lupus patients have been reported to exhibit a depletion of intracellular glutathione, a vital cellular antioxidant [129]. Glutathione depletion has been reported to increase the target of the rapamycin (mTOR) signaling pathway [130,131]. The mTOR signaling pathway acts as a central regulator in cell metabolism, growth, proliferation and survival by mainly controlling energy utilization and protein synthesis, and this pathway exists in almost all immune cells [132,133]. Activation of mTOR occurs in T cells and other cell types in lupus and is responsible for multiple pathogenic processes [134,135,136,137,138]. Recent studies have suggested that the addition of N-acetyl L-cysteine (NAC), which helps to replenish intracellular glutathione, decreases lupus through blocking mTOR [139]. 

The kynurenine pathway is a metabolic pathway leading to the production of nicotinamide adenine dinucleotide (NAD+) from the degradation of the essential amino acid tryptophan. Disruption in the pathway is associated with certain genetic disorders [140,141,142]. In lupus patients, it was reported that NAC significantly reduced kynurenine, which also decreased mTOR signalling. These results suggest that reversal of glutathione depletion by the amino acid precursor or inhibition of the kynurenine pathway may reduce the activation of mTOR in SLE [130,143,144]. Rapamycin, a specific mTOR inhibitor, can effectively decrease lupus disease in both lupus-prone mice and patients [135]. Rapamycin was shown to promote demethylation of genes at the 5-position of cytosine (5mC) in the mTOR pathway in naïve CD4+ T cells. This inhibited the differentiation of Th1 and Th17 cells that can both contribute to lupus development [145].

The activation of mTOR promotes glycolysis and lipogenesis to generate acetyl-CoA, which the substrate histone acetylation relies on to provide an acetyl group and complete the reaction. Indeed, one of the targets of mTOR is the acetylation of histone proteins. However, the consequence of this is not clear. In one study, it was shown that acetylation of histone H3 at lysine 56, H3K56ac, was directly inhibited by rapamycin [146,147]. However, H3K56ac was activated by mTOR and promoted mTOR-dependent growth, suggesting positive feedback between mTOR activation and histone acetylation [148]. Furthermore, histone deacetylation is mediated by the nicotinamide adenine dinucleotide (NAD) dependent sirtuin deacetylase. The bio-generation of NAD from the kynurenine pathway is upregulated in lupus. Additionally, it has been shown that the kynurenine pathway also promotes mTOR activation which, as discussed above, promotes histone acetylation [142,149,150,151]. Therefore, an overall balance and availability of metabolic substrates may determine opening or closing of gene transcriptions by affecting the level of histone acetylation or deacetylation, respectively.

## 6. Summary

Aberrant epigenome gene regulation and modification plays a crucial role in the pathogenesis of SLE. Comprehensive understanding of how epigenetic modification and acetylation/deacetylation of non-histone proteins corrects or promotes autoimmune disease will enable us to gain further insight into the pathogenic mechanisms of autoimmune disorders. By understanding how acetylation/deacetylation and methylation/demethylation modulate gene expression and cell signaling, we will be able to more effectively target the signaling cascades and gene expressions that initiate and promote aberrant cell function in SLE.

## Figures and Tables

**Table 1:** Summary of HDAC Classifications.

Histone Deacetylases (HDAC) Classification	Enzymatic Activity	Mechanism of Action	Location	Substrates	HDAC Inhibitor	Autoimmunity and Systemic Lupus Erythematosus (SLE) Involvement
**Class I.**						
HDAC1	Enhanced when incorporated into complexes	1 class I catalytic domain	Nucleus	p53, RB, MyoD, NF-kB, DNMTI, DNMT3a, MBD2, Sp1, BRCA1, MeCP2, ATM, Smad7 [61,92]	Valproic acid, phenylbutyrate, MS-275, Romidepsin, Suberoylanilide Hydroxamic Acid [93]	Overexpression of HDAC1 increases the activity of the 3’-IgH enhancers. HDAC1 is recruited to the IgH enhancer region, and TSA treatment of B cells reduced the production of anti-DNA autoantibodies.
HDAC2	Enhanced when incorporated into complexes	1 class I catalytic domain	Nucleus	RB, NF-kB, BRCA1, DNMTI [61]	Valproic Acid, phenylbutyrate, Suberoylanilide Hydroxamic Acid, MS-275, Romidepsin [93,94,95]	Critical for transcriptional regulation, cell cycle progression and developmental processes.
HDAC3	Enhanced when incorporated into complexes	1 class I catalytic domain	Nucleus/Cytoplasm	RB, NF-kB, Smad7, Stat3, SRY [61]	Valproic Acid, Suberoylanilide Hydroxamic Acid, MS-275 [93,96]	HDAC3 gene expression is decreased in SLE monocytes, involved in macrophage polarization.
HDAC8	Fully active in isolation	1 class I catalytic domain	Nucleus	Not Reported	Suberoylanilide Hydroxamic Acid, Resveratrol, APHA, Curcumin [93,97]	Downregulate the expression of pro-inflammatory cytokines (TNF-alpha, TGF-beta, IL-1beta, and IL-6).
**Class IIa.**						
HDAC4	Weak enzymatic activity in isolation	1 class II catalytic domain	Nucleus/Cytoplasm	GCMa, GATA-1, HP-1 [92,98,99]	Not reported	Role in pro-inflammatory gene expression.
HDAC5	Weak enzymatic activity in isolation	1 class II catalytic domain	Nucleus/Cytoplasm	GCMa, Smad7, HP-1 [92,100]	TSA [93]	HDAC5 mRNA expression is enhanced in inflammatory states.
HDAC7	Weak enzymatic activity in isolation	1 class II catalytic domain	Nucleus/Cytoplasm	PLAG1, PLAG2 [92,101]	Not reported	Promotes inflammatory responses in macrophages, regulates TLR responses in macrophages, regulates LPS signaling.
HDAC9	Weak enzymatic activity in isolation	1 class II catalytic domain	Nucleus/Cytoplasm	Not Reported	Suberoylanilide Hydroxamic Acid, MS-275 [93]	Regulates Foxp3-dependent suppression. Increase in Treg cells—decrease in suppressive activity. HDAC9 inhibition may benefit SLE patients as shown in MRL/lpr mice.
**Class IIb.**						
HDAC6	Acts on structural proteins	2 class II catalytic domains with 1215 amino acids. SE14 repeats. BUZ is ZnF domain	Mainly cytoplasmic	Smad7, α-Tubulin, Hsp90 [61,102]	M344 [92,93,103]	HDAC6 is overexpressed in SLE—causes an increased B cell development and response. Inhibition causes reduced germinal center B cells, T follicular cells and IFN-gamma secreting cells.
HDAC10	Not measurable	2 class II catalytic domains	Nucleus/Cytoplasm	Not reported	Not reported	Overexpressed in B cells from the spleen.
**Class IV.**						
HDAC11	Regulates immune activation and immune tolerance	1 class IV catalytic domain	Nucleus	Not reported	Not reported	Gene expression is decreased in SLE monocytes, negative transcriptional regulator

## Abbreviations

SLE	Systemic lupus erythematosus
MHC	Major histocompatibility complex
HDACs	Histone deacetylase
HATs	Histone acetyltransferase
MEF2	Myocyte enhancer factor 2
TFN I/II	Type I/II interferons
TNF	Tumor necrosis factors
BLys	B-lymphocyte stimulators
LN	Lupus nephritis
SNPs	Single-nucleotide polymorphisms
TLR	Toll like receptor
SLEDAI	SLE disease activity index
TSA	Trichostatin
SAHA	Suberonylanilide hydroxamix acid
PBMC	Peripheral blood mononuclear cell
NZB/W	New Zealand Black/White
